# Factors influencing success in quality-improvement collaboratives: development and psychometric testing of an instrument

**DOI:** 10.1186/1748-5908-5-84

**Published:** 2010-10-28

**Authors:** Loes MT Schouten, Richard PTM Grol, Marlies EJL Hulscher

**Affiliations:** 1 Dutch Institute for Healthcare Improvement, Utrecht, The Netherlands; 2 Nijmegen Medical Centre, Radboud University, Nijmegen, The Netherlands

## Abstract

**Background:**

To increase the effectiveness of quality-improvement collaboratives (QICs), it is important to explore factors that potentially influence their outcomes. For this purpose, we have developed and tested the psychometric properties of an instrument that aims to identify the features that may enhance the quality and impact of collaborative quality-improvement approaches. The instrument can be used as a measurement instrument to retrospectively collect information about perceived determinants of success. In addition, it can be prospectively applied as a checklist to guide initiators, facilitators, and participants of QICs, with information about how to perform or participate in a collaborative with theoretically optimal chances of success. Such information can be used to improve collaboratives.

**Methods:**

We developed an instrument with content validity based on literature and the opinions of QIC experts. We collected data from 144 healthcare professionals in 44 multidisciplinary improvement teams participating in two QICs and used exploratory factor analysis to assess the construct validity. We used Cronbach's alpha to ascertain the internal consistency.

**Results:**

The 50-item instrument we developed reflected expert-opinion-based determinants of success in a QIC. We deleted nine items after item reduction. On the basis of the factor analysis results, one item was dropped, which resulted in a 40-item questionnaire. Exploratory factor analysis showed that a three-factor model provided the best fit. The components were labeled 'sufficient expert team support', 'effective multidisciplinary teamwork', and 'helpful collaborative processes'. Internal consistency reliability was excellent (alphas between .85 and .89).

**Conclusions:**

This newly developed instrument seems a promising tool for providing healthcare workers and policy makers with useful information about determinants of success in QICs. The psychometric properties of the instrument are satisfactory and warrant application either as an objective measure or as a checklist.

## Introduction

Approaches to collaborative quality improvement currently form one of the most popular methods for organising improvement in hospitals and ambulatory practices. A quality-improvement collaborative (QIC) is an approach emphasising collaborative learning, support, and exchange of insights among different healthcare organisations. It brings together multidisciplinary teams from different organisations and agencies that share a commitment to making small, rapid tests of change that can be expanded to produce breakthrough results in a specific clinical or operational area [1]. Although the underlying basic concept of QIC programmes appears intuitively appropriate, QICs have not been linked to a published evidence base of effectiveness [2]. A recent systematic review of QICs showed moderately positive results and varying success in achieving collaborative goals [3]. Insight into the mechanisms responsible for the results and variation in a QIC is scarce [4].

While unequivocal evidence of the effectiveness of the method may be lacking, QIC approaches have been initiated worldwide, and they represent substantial investments of time, effort, and funding in the healthcare delivery system [5]. Given the popularity of collaborative approaches, it seems obvious that future designers and implementers of collaboratives should be guided by information on how to optimize the benefits of QICs. This requires a better understanding of the factors that determine their success.

Although a few studies have explored the presence of conditions for successful implementation of collaboratives [6-9], an analysis of theoretical concepts influencing the impact of QICs is absent, as is an overview of the key characteristics of the approach relating to success. Moreover, sound information as to why particular QICs worked in specific settings, organisations, or teams but not in others and what factors influenced their success or lack of success are likewise absent. One step in gaining such an understanding is a comprehensive, valid, and reliable measurement of such factors. We have therefore developed and tested a new tool to measure factors that might influence success in QICs. This instrument can be used as a measurement instrument to collect information about perceived determinants of success retrospectively. In addition, it can be applied prospectively as a checklist to guide initiators, facilitators, and participants of QICs, with information about how to carry out or participate in a collaborative with theoretically optimal chances of success. Such information can be used to evaluate and improve QIC approaches.

## Methods

The instrument was developed in several steps.

### Developing an instrument with content validity

'Factors influencing success in a QIC' is the focal construct of this QIC instrument. To increase confidence that the instrument measures the aspects it was designed for, we addressed content validity according to published procedures [10]. The aim was to ensure that the instrument content was relevant and thoroughly represented the potential determinants of success in QICs. The first step we took to distinguish and define potential determinants of success in a QIC was to use a systematic search [3] to find theoretical papers about QICs. We searched the MEDLINE^® ^(US National Library of Medicine, Bethesda, MD, USA), CINAHL^® ^(EBSCO Publishing, Ipswich, MA, USA), Embase^® ^(Elsevier B.V., New York, NY, USA), Cochrane, and PsycINFO^® ^(American Psychological Association, Washington, DC, USA) databases for literature about QICs in the period from January 1995 to June 2006, inclusive. We started with a MEDLINE search for free text terms describing QICs, and we combined the keywords (non-MeSH) 'quality and improvement and collaborative' or '(series or project) and breakthrough'. The same steps were repeated for the other databases. We also reviewed the reference lists of the included papers. To distinguish and define determinants of success, studies were included if they (a) gave an overview of key elements or components of QICs applied in healthcare and (b) were written in English. Two researchers (LS and MH) reviewed titles of articles and abstracts identified in the search. Each potentially eligible paper was independently assessed. The reference lists of the papers were also reviewed.

Our search identified five studies that met our inclusion criteria [1,11-14]. All authors were experts in the field of QICs. Two reviewers (LS and MH) independently extracted the characteristics of the collaboratives and the theoretical concepts influencing success from these papers. Then they categorized the items using the following definition as a template: 'A QIC is an organised, multifaceted approach to quality improvement that involves five essential features, namely, (1) there is a specified topic, (2) clinical experts and experts in quality improvement provide ideas and support, (3) multiprofessional teams from multiple sites participate, (4) there is a model for improvement (setting targets, collecting data, and testing changes), and (5) the collaborative process involves a series of structured activities'[3].

The five papers with an overview of collaboratives provided a list of 128 items of expert-opinion-based determinants of success [15]. Two reviewers (LS and MH) analysed the list of determinants to identify problems with wording or meaning and redundancy or relevancy of items. Items measuring similar determinants were categorized together. Determinants with potential overlap in construct and those that were deemed vague, ambiguous, or redundant were removed. This exercise reduced the list to 72 items.

After revisions of wording and sequencing of questions, four experts involved in QICs reviewed the first draft of the instrument to enhance the face validity. They were asked to judge the questions for readability, comprehensibility, ease of response, and content validity. After review by the expert panel, the list was reduced to 50 items. Overall, the reviewers' responses were similar in nature, with no noteworthy variance. As part of the content validity testing, items were accepted or deleted on the basis of the level of agreement between the reviewers, and appropriate changes were made in accordance with the suggestions of the experts. As a result, the QIC instrument was thoroughly critiqued and refined [16].

The 50-item instrument that was created was intended to represent four subscales believed to represent various determinants of success in a specific QIC: (1) sufficient expert panel support, (2) effective multiprofessional teamwork, (3) appropriate use of the improvement model, and (4) helpful collaborative processes. A five-point Likert scale was used in the design of the items and ranged from strongly disagree to strongly agree.

### Testing the instrument

#### Sample and data collection

To comprehensively test the construct validity and the internal consistency of our QIC instrument, we asked participants in current national collaboratives to complete the instrument. Our sample represented healthcare workers from 46 multidisciplinary quality improvement teams participating in two distinct collaboratives based on the Breakthrough Series [12], one focusing on breast cancer and one on perioperative care. Each team consisted of a minimum of four people. Individual team members were asked to complete the questionnaire at the last conference or post completed questionnaires to us. In order to examine the central tendency, variability, and symmetry, we calculated descriptive statistics and the response distribution for each item. To enhance feasibility, we considered reducing the number of items. Items with the following characteristics were removed: those with a high proportion of missing responses (> 10%), those that showed redundancy of measurement through a high correlation (*r *> .85) with another item, and those with skewed distributions (items with > 90% of the answers in categories 1 and 2 or 4 and 5 on a five point likert scale).

Before items were removed, their importance was considered, as judged by the reviewers' (LS and MH) opinions of their content validity.

#### Construct validity testing: Exploratory factor analysis

We used principal components analysis for the exploratory factor analysis to analyse the construct validity, defined as the extent to which a test measures a theoretical construct or trait [17,18]. We used SPSS 16.0^® ^(IBM, Chicago, IL, USA) to select the final items for the questionnaire. We used a maximum likelihood solution with varimax, an orthogonal rotation method that minimizes the number of variables with high loadings on each factor. This method simplifies the interpretation of the factors. A precedent cutoff of 0.4 was specified for acceptable factor loadings, and items with a loading of 0.4 or more were retained [19].

### Internal consistency testing

#### Internal homogeneity

We used Cronbach's alpha to measure the internal homogeneity, defined as the extent to which subscales of an instrument measure the same attribute or dimension. Internal homogeneity represents an index of an instrument's reliability [20,21].

As the QIC instrument was an assembly of items in four subscales designed to quantify agreement with the determinants of success in a QIC, it was important to know whether the set of items in the subscales consistently measured the same construct. For the purposes of this study, a Cronbach's alpha of .7 or more was considered acceptable for the composite scores on the subscales of the QIC instrument as a self-report instrument [22]. Data acquired from the collaborative participants were used to test internal consistency. Underlying theoretical constructs suggested that a positive correlation should be expected between all items in a subscale.

#### Intercorrelations

To test item-internal consistency, the correlations of the items with their scales were determined. High convergent validity of the items was indicated if the item correlated with the relevant scale. A matrix was set up with item-scale correlations comparing correlations across scales.

## Results

### Sample

All 46 established improvement teams participated in the working conferences (learning sessions) and completed the collaborative. There were no dropouts. The mean number of team members was 7 (range: 4 to 13), although not all team members attended the conferences. All teams included at least one medical specialist, one nurse, and one allied health professional. Representing 44 teams, 144 participants attending the last conference completed the questionnaire (response rate: 95%). The numbers of valid responses were high for all items, providing evidence that items and response choices were clear and unambiguous. Table 1 displays the descriptive statistics of the items. Both collaborative topics (breast cancer and perioperative care) showed high scores (mean scores ≥4) for the presence of more than half of the potential determinants. Most items showed little variation (the standard deviation varied between 0.515 and 1.17). No items were excluded on the basis of the proportion of missing responses. We deleted nine items from the initial 50-item instrument with 90% of the answers in categories 4 and 5: 1.3 (chairperson was an expert), 2.10 (general goals of the collaborative were clear), 2.11 (team supported collaborative's general goals), 2.15 (team directly involved in changes), 2.16 (team had relevant expertise), 2.18 (teams were motivated), 2.21 (team focused on patient improvement), 2.22 (team focused on care process improvement), 3.28 (team gathered measurement data),

**Table 1:** Item-descriptive statistics of the questionnaire

Items	Mean	SD
**Sufficient expert panel support**		
1.1 The collaborative chairperson was an opinion leader	4.10	0.697
1.2 The expert panel provided information and advice for changes	4.11	0.655
1.3 The collaborative chairperson was an expert on the QIC topic	4.45	0.686
1.4 The expert panel provided sufficient time for our project	4.03	0.687
1.5 The expert panel provided positive feedback for our project	3.95	0.702
1.6 The expert panel was experienced in successfully improving the care process for the QIC topic	4.09	0.758
1.7 The expert panel contributed scientific knowledge	4.25	0.742
1.8 The expert panel contributed practical experience	4.18	0.778
**Effective multidisciplinary teamwork**		
2.9 Collaborative participation was carefully prepared and organised	3.84	0.894
2.10 General goals of the collaborative were clear	4.29	0.549
2.11 My team supported the collaborative's general goals	4.29	0.617
2.12 Management provided sufficient means and time	3.48	1.170
2.13 Management followed project progress	3.22	1.115
2.14 Management prioritised success	3.37	0.963
2.15 Team members were directly involved in changes	4.37	0.600
2.16 Team members had relevant expertise	4.41	0.539
2.17 Team members had leadership skills	4.12	0.794
2.18 Teams were motivated in implementing changes	4.19	0.637
2.19 Roles in my team were clearly defined	3.93	0.755
2.20 Participation in this project enhanced multidisciplinary collaboration in my organization	4.15	0.743
2.21 My team focused on patient improvement	4.31	0.572
2.22 My team focused on care-process improvement	4.26	0.565
**Appropriate use of the improvement model**		
3.23 My team formulated clear goals	4.02	0.737
3.24 My team focused on achieving goals	4.05	0.719
3.25 Goals were discussed within organisation	3.71	0.805
3.26 Goals were incorporated in organisation policy	3.84	0.768
3.27 Goals were readily measurable	4.04	0.669
3.28 My team gathered measurement data	4.36	0.585
3.29 My team used measurements to plan changes	3.93	0.862
3.30 My team used measurements to test changes	3.68	0.996
3.31 My team used measurements to track progress	4.11	0.734
3.32 My team considered continuous improvement a part of working process	3.91	0.699
3.33 My team continued to aim for change	3.63	0.802
3.34 My team tracked progress continuously	3.80	0.754
**Helpful collaborative processes**		
4.35 Useful knowledge and skills we given to my team during working conferences	3.88	0.699
4.36 Focus was on practical application of knowledge and skills at working conferences	3.78	0.651
4.37 My team shared experiences at working conferences	4.05	0.587
4.38 Working conferences focused on joint learning	3.95	0.656
4.39 My team developed skills in planning changes at working conferences	3.68	0.752
4.40 My team developed skills in processing changes at working conferences	3.66	0.756
4.41 My team developed confidence in achievability of changes at working conferences	3.88	0.721
4.42 Teams reflected on results at working conferences	4.05	0.515
4.43 My team contacted coworkers from other organisations at working conferences	3.77	0.815
4.44 My team learned from progress reporting by other teams at working conferences	3.92	0.659
4.45 Teams received feedback on progress from expert panel at working conferences	3.72	0.720
4.46 Teams supported one another at working conferences	3.49	0.774
4.47 There was competition between teams during the joint working conferences	2.74	0.996
4.48 There was a moment to reflect on achieved results	3.96	0.607
4.49 Information, ideas, and suggestions were actively exchanged at working conferences	3.65	0.694
4.50 Teams exchanged information outside working conferences	2.73	0.968

SD = standard deviation; QIC = quality improvement collaborative.

### Construct validity testing: Exploratory factor analysis

**Figure 1:**
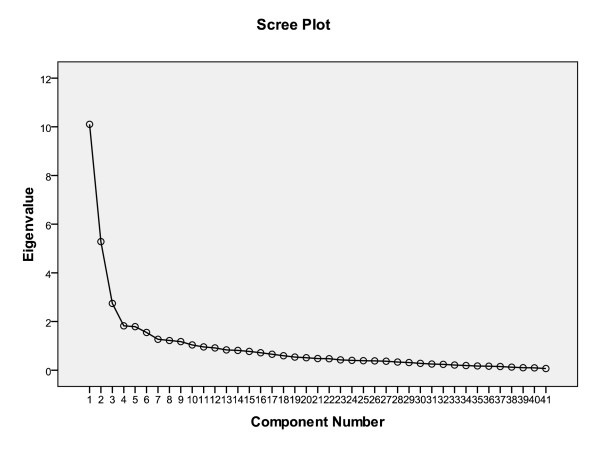
**Scree plot**.

Exploratory factor analysis showed the 50 items to be clustered in three scales (Figure 1). Together, these three accounted for 44.2% of the total variance. Table 2 presents the items of the scales and their factor loadings for the three-factor solution, after varimax rotation. Item 4.47 (there was competition between improvement teams at the joint working conferences) was removed because the factor analysis showed it did not fit with any distinct factors representing the different concepts. It was not necessary to apply a second criterion; none of the remaining items loaded on more than one factor after varimax rotation.

**Table 2:** Factor loadings for the list for the quality improvement collaborative

**Rotated component matrix**^**a**^			
Item	Component		
	1	2	3
1.8 Expert panel contributed practical experience			0.755
1.7 Expert panel contributed scientific knowledge			0.741
1.6 Expert panel was experienced in successfully improving care process			0.725
1.2 Expert panel gave advice on changes			0.676
1.1 Chairperson of the expert panel was an opinion leader			0.627
1.4 Expert panel had ample time			0.617
1.5 Expert panel gave positive feedback			0.611
			
			
3.23 My team formulated clear goals	0.747		
2.19 Roles in my team were clearly defined	0.731		
3.24 My team focused on achieving goals	0.728		
3.32 My team considered continuous improvement a part of working process	0.718		
2.09 Collaborative participation was carefully prepared and organized	0.705		
3.34 My team tracked progress continuously	0.690		
2.17 Team members had leadership skills	0.658		
3.27 Goals were readily measurable	0.652		
2.14 Management prioritised success	0.639		
2.12 Management provided sufficient means and time	0.605		
3.25 Goals were discussed within organization	0.530		
3.33 My team continued to aim for change	0.527		
2.20 Participation in this project enhanced multidisciplinary collaboration in my organisation	0.521		
3.29 My team used measurements to plan changes	0.521		
2.13 Management followed project progress	0.514		
3.30 My team used measurement to test changes	0.511		
3.31 My team used measurements to track progress	0.487		
3.26 Goals were incorporated in organisation policy	0.483		
			
4.40 My team developed skills in processing changes at working conferences		0.732	
4.39 My team developed skills in planning changes at working conferences		0.711	
4.44 My team learned from progress reporting by other teams at working conferences		0.668	
4.38 Working conferences focused on joint learning		0.654	
4.36 Focus was on practical application of knowledge and skills at working conferences		0.651	
4.43 My team contacted coworkers from other organisations at working conferences		0.645	
4.46 Teams supported one another at working conferences		0.628	
4.49 Information, ideas, and suggestions were actively exchanged at working conferences		0.623	
4.35 Useful knowledge and skills were given to my team during working conferences		0.617	
4.48 There was a moment to reflect on achieved results		0.561	
4.37 My team shared experiences at working conferences		0.558	
4.41 My team developed confidence in achievability of changes at working conferences		0.511	
4.50 Teams exchanged information outside working conferences		0.509	
4.45 Teams received feedback on progress from expert panel at working conferences		0.509	
4.42 Teams reflected on results at working conferences		0.487	

^a^Rotation converged in five iterations.

Extraction method: principal component analysis; rotation method: varimax with Kaiser normalization; item excluded: 4.47: There was competition between teams during the joint working conferences.

Overall, all items from the scale 'clinical experts and experts in quality improvement provide ideas and support for improvement' (seven items) and 'the collaborative process involves structured activities' (15 items) loaded on their theoretical scales. The original scales 'multiprofessional teams from multiple sites participate' and 'use of a model for improvement' converged (in total, 18 items). The three components were labeled: 'sufficient expert panel support', 'effective multidisciplinary teamwork', and 'helpful collaborative processes'.

### Internal consistency testing

#### Internal homogeneity

Cronbach's alpha analysis of the three scales revealed alphas between .85 and .89, which indicates very good reliability for all three factors of the instrument.

#### Intercorrelations

All factors or scales correlated significantly and positively (Table 3). Scale correlations ranged from .205 ('sufficient expert panel support' and 'effective multidisciplinary teamwork') to .398 ('helpful collaborative process' and 'effective multidisciplinary teamwork'). The inter-item correlations show adequate levels of inter-scale correlations (Table 4).

**Table 3:** Correlations calculated as Spearman's rho

		Support from expert team	Multidisciplinary team, improvement model	Collaborative process
Sufficient expert panel support	Correlation coefficient	1.000		
	Significance (two-tailed test)			
Effective multidisciplinary teamwork	Correlation coefficient	.230*	1.000	
	Significance (two-tailed test)	.050	.	
Helpful collaborative processes	Correlation coefficient	.410**	.323**	1.000
	Significance (two-tailed test)	.000	.004	

*Correlation is significant at the .05 level (two-tailed test); **Correlation is significant at the .01 level (two-tailed test).

**Table 4:** Intercorrelations and reliabilities among scales

	Items	Alpha coefficient	Interitem correlation (lowest to highest)	Interscale correlation		
Scale				1	2	3
1. Sufficient expert panel support	7	.85	.255-.712			
2. Effective multidisciplinary teamwork	18	.89	.046-.777	.205		
3. Helpful collaborative processes	15	.88	.132-.834	.388**	.398**	

## Discussion

This study comprehensively explored the potential determinants of success that can be included in measuring the impact of QICs. The theoretical framework of our instrument was exclusively built on information from literature and expert opinion concerning QICs. We based our instrument on four key components of QICs: (1) clinical experts and experts in quality improvement provide ideas and support for improvement, (2) multiprofessional teams from multiple sites participate, (3) there is a model for improvement (setting targets, collecting data, and testing changes), and (4) the collaborative process involves a series of structured activities. We would expect that factors reflecting any of these key components potentially influence the success or failure of QICs. For example, 'expert panel support' may play an important role in legitimizing the collaborative and motivating the participants. Effective 'multiprofessional teamwork' may require gathering the right individuals for an improvement team, committing to change, and securing time, resources, and management support. Engaging in a 'model for improvement' is assumed to build the internal capacity of participating organisations to establish clear aims, to collect and monitor appropriate performance measures, and to set the stage for continuous improvement. Finally, 'collaborative processes and activities' are targeted to enable mutual learning, social comparison, and support. The factor structure found in the data is almost identical to the four subcategories we theorised. However, 'multiprofessional teams' and 'there is a model for improvement' loaded on one factor. Rather than four, we found three factors in exploratory factor analysis. Items reflecting internal-team features, like multiprofessional teamwork, senior management support, and clarity of roles, coincided with features like setting aims, collecting data, and testing changes, at least in the eyes of the QIC participants.

Duckers *et al*. [6] developed a 15-item instrument for team organisations and supportive conditions to implement QIC projects using literature about QICs, team-based implementation, and the dissemination of innovations within health service organisations. Mills *et al*. [7,8] and Neilly *et al*. [9] used surveys based on research in team performance and organisational learning and the characteristics of high-performing healthcare microsystems to assess determinants of success in QICs. While some items in these instruments overlap with ours (*e.g.*, items reflecting teamwork, leadership and/or organisational support), several differences remain (Table 5). Our instrument was built exclusively on the key components of QICs based on expert literature and expert opinion about QICs. With the exception of the feature 'there is a specified topic' (excluded from our instrument as a prerequisite assumed not to vary in one specific QIC), our instrument reflects the key components of a collaborative, adding items about the use of opinion leaders as change agents; setting clear and measurable goals; multidisciplinary collaboration; receiving feedback on progress; reflecting on results at working conferences; and focusing on sharing, exchanging, joint learning, and external peer support.

**Table 5:** Overview of questionnaire scales

Questionnaire scales		
**Mills *et al*. (2003; 2004); Neily *et al*. (2005) **[7-9]	**Duckers *et al*. (2008) **[6]	**Schouten *et al*. (2010)**

Leadership support	Organisational support	Sufficient expert panel support

Teamwork skills	Team organisation	Effective multidisciplinary teamwork

Prior experience with quality improvement and teamwork.	External change agency support	Helpful collaborative processes

New skills, information exchange, and overall satisfaction		

Useful information systems		

Although only in the first stages of development and validation, our instrument seems a promising tool that will be able to provide healthcare workers, facilitators, managers, and researchers with a more specific understanding of success determinants in approaches to collaborative quality improvement. Participant completion of the QIC instrument during or after the QIC will provide researchers, healthcare workers, facilitators, and managers with an objective measure of the perceived success of determinants in a QIC. In addition, with a little rephrasing, the instrument can be applied as a checklist to prospectively guide initiators and facilitators of a QIC by providing information on how to carry out a collaborative with theoretically optimal chances of success. This information can be used to adapt the performance of the QIC during (for current participants) or after (for future participants) the QIC. Thus, hospital managers, project teams, external change agents, researchers, and other interested public parties may benefit from this instrument since it provides ready information relevant to real-time adjustments, intake procedures, and further research.

### Limitations

Our testing has some limitations. First, a few remarks must be made with regard to the sample size. Different standards are applied for the number to cases ratio of items for a factor analysis versus a principal component analysis. Five to ten cases for each item are generally recommended [23,24]. Others state that the most important issues in determining reliable factor solutions are the absolute sample size and the absolute magnitude of factor loadings. For example, Guadagnoli and Velicer [25] state that a factor with four or more loadings greater than 0.6 is reliable, regardless of sample size. In our analysis, 7 out of 7 (factor 1), 10 out of 18 (factor 2), and 9 out of 15 items (factor 3) showed loadings > 0.60.

Second, we were unable to test the temporal reliability, so we could not compute a test-retest reliability coefficient and did not assess the discriminating capacity. Third, we tested our instrument by using it as a measurement instrument to retrospectively collect information about perceived determinants of success. Appropriately applying the instrument prospectively (as a checklist) may require the same steps as for testing construct validity and internal consistency. Finally, the relatively high scores of the 44 multidisciplinary improvement teams that completed the instrument in this study do suggest that most determinants or conditions in these specific collaboratives were present or fulfilled. These scores are not necessarily applicable to other teams or QIC initiatives. As participating teams vary in their individual performance and amount of improvement, further research is needed to quantitatively determine its usefulness in explaining the differences of success between teams participating in a QIC.

Many experts and researchers involved in QICs have pointed out that it would be helpful to understand which success factors are associated with outcomes in QICs. It is therefore important to have access to assessment tools that have undergone evaluation and have been proven to be valid and reliable. This study shows that the psychometric properties of this newly developed instrument are satisfactory. Further research to refine the instrument and link its outcomes to key effect parameters is needed to estimate its usefulness in quantitatively explaining the differences of success in a QIC.

## Competing interests

The authors declare that they have no competing interests.

## Authors' contributions

LMTS participated in the design of the study, carried out the data collection and performed the statistical analysis. MEJHH and RPTMG conceived of the study, and participated in its design and coordination and helped to draft the manuscript. All authors had full access to all of the data (including statistical reports and tables) in the study and take responsibility for the integrity of the data and the accuracy of the data analysis. All authors read and approved the final manuscript.
